# Congenital Displacement of the Head of the Femur and Its Treatment

**Published:** 1894-05-12

**Authors:** A. H. Tubby

**Affiliations:** Surgeon to Out-patients, Evelina Hospital for Sick Children; Surgeon, National Orthopædic Hospital; Senior Demonstrator of Physiology, Guy's Hospital


### Medical Progress and Hospital Clinics.

[*The Editor will be glad to receive offers of co-operation and contributions from members of the profession. All letters should be addressed to* The Editor, The Lodge, Porchester Square, London, W.]

### CONGENITAL DISPLACEMENT OF THE HEAD OF THE FEMUR AND ITS TREATMENT.

By A. H. Tubby, M.S.Lond., F.R.C.S.Eng., Surgeon to Out-patients, Evelina Hospital for Sick Children; Surgeon, National Orthopædic Hospital; Senior Demonstrator of Physiology, Guy's Hospital.

This morbid condition in the lower extremity is often incorrectly called congenital dislocation of the hip, but the study of the pathology of veritable cases proves this to be a misnomer, since in such instances there is no real or perfect acetabulum from which the head of the femur can be dislocated.

Now under these headings several conditions not congenital are included. Unskilful or violent delivery, especially in breech presentations resulting in dislocation at the moment of birth, cannot be classified as congenital. That such accidents may readily happen is proved by some experiments by Melicher in Vienna upon women who had died undelivered. In six trials he found that with the fingers or crotchet it was possible to produce the luxation, and it occurred in two instances, both unilateral, and in that hip to which greater traction had been applied as the more immediately presenting part. Reduction in these cases was effected with the greatest difficulty. I am always inclined, if the displacement be unilateral, and the presentation pelvic, and if the labour were a difficult one, to suspect that the condition occurred during birth. And this suspicion is strengthened if at the time of birth there were noted signs of effusion swelling, and hæmatoma.

Among the supposed causes of true congenital displacement is malposition *in utero*, and it is somewhat curious to note that many such cases have been breech presentations. But with Bowlby, I believe it is not possible for displacement to occur when the thighs are flexed on the abdomen in pelvic presentations, if the cotyloid cavity be perfect. Various intra-uterine pathological conditions, supposititious, or real, have been invoked to explain the occurrence of this deformity. Such, for instance, are a supposed lesion of the nerve centres, contraction or paralysis of certain muscles, laxity of the ligaments, and intra-uterine coxitis. But unfortunately, with the exception of the first and last, which are chimerical, these are results and not causes.

For the true origin we must look to failure in development. The normal development of the hip joint commences on the 22nd or 23rd day, when the lower limb of the embryo appears merely as a bed of mesoblast; then chondrification of the cells to form the femur and pelvis takes place, but between them is a layer of mesoblast, which stains deeply with reagents, and forms the soft tissues of the joint. The hip joint is not at first a socket, but merely a spot on the bone, on which the head of the femur lies. Around this the acetabulum grows up in three distinct portions, the iliac, ischial, and pubic, and forms a deep cup by the end of the third month, which develops into a perfect acetabulum.

The general appearances in displacement have been well described by Bowlby, Lockwood, and Adams in the Pathological Society *Transactions*. The head of the femur is unaltered in shape and covered with cartilage. It may be displaced upwards, upwards and forwards, or upwards and backwards. In my experience it is frequently upwards and forwards, although this may be a secondary position. The capsular ligament is enormously thickened, especially at its upper part, and elongated. There is deficiency of the acetabulum. The iliac portion is frequently absent. Hence the cavity is triangular and not round, and it is impossible for the head to fit into it. The rim of the acetabulum is deficient above and behind, and the bone passes upwards and backwards. The sequence of events probably is imperfect development of the iliac portion of acetabulum, slipping away of head of femur, and formation of a triangular shaped acetabulum, into which it is impossible for the head to be replaced. Above the acetabulum is a spot on which the head of the bone rests. The round ligament is as often present as absent. Unfortunately, the recognition of the abnormal condition is not often made till the time when the child should walk. But early diagnosis is important for the following reasons:—(*a*) As to the possibility of cure, treatment should be begun at eighteen months to two years, *i.e.*, before the child walks; (*b*) to avoid mistaken treatment, such as for paralysis; (*c*) lest unjust accusations be made against nurses and those in charge of the child, and the condition be referred to a supposed fall or other neglect.

The symptoms of true congenital displacement upon which we mainly rely are the approximation of the trochanter to the crest of the ilium, traction on the limb drawing it down temporarily, the peculiar waddling gait, the lordosis, the want of proportion between the upper and lower limbs, and the excessively free and painless movements. A bilateral condition is usually truly congenital.

What is the outlook? If the case be left without treatment it goes on from bad to worse, until walking is well nigh impossible. If the case be carefully treated on conservative lines very much good may be effected, and the deformity may be greatly reduced, or even cured. At the present moment I have under my care a boy, aged seven, on whom I am in good hopes of effecting a permanent cure. The method I have adopted is that of complete extension for eighteen months to two years on the lines so successfully laid down by Buckminster Brown and William Adams, followed by the use of appropriate walking instruments and crutches for the succeeding two years. What is it I hope will be effected? Not that the head will become settled in its proper place, the acetabulum. This we have shown to be impossible, on account of the shape of the latter. But rather that the elongated capsule will be much shortened, and being already thickened, especially at its upper part, it will prove sufficiently strong to retain the head of the bone close to its natural position. At the same time, the external rotators and other muscles will have entirely lost their spasm on account of the prolonged rest, and will no longer tend to displace the head of the femur with every step.

The injection of solutions of chloride of zinc has been favourably spoken of, and I can well conceive that, if made with all proper precautions, they will set up sufficient irritation in the neighbourhood of the head of the femur to consolidate it firmly in its new position, I should advocate an extensive trial of this method as an adjunct to prolonged rest, and at the end of a year and a-half of complete recumbency.

In true congenital dislocations attempts to make new acetabula, or new joints on the mortice principle, have proved signal failures.

